# Young people’s tech identity performances: why materiality matters

**DOI:** 10.1186/s40594-020-00249-w

**Published:** 2020-10-06

**Authors:** Spela Godec, Uma Patel, Louise Archer, Emily Dawson

**Affiliations:** 1 Department of Education, Practice and Society, UCL Institute of Education, University College London, 20 Bedford Way, London, WC1H 0AL UK; 2 UCL Department of Science and Technology Studies, University College London, 22 Gordon Square, London, WC1H 0AW UK

**Keywords:** Identity performativity, Technology, Materiality, Intra-action, Barad, Butler

## Abstract

**Background:**

Identity provides a useful conceptual lens for understanding educational inequalities in science, technology, engineering and mathematics (STEM). In this paper, we examine how paying attention to physical and digital ‘materiality’ enriches our understanding of identity work, by going beyond the spoken, written and embodied dimensions of identity performances that currently dominate the area of STEM identity scholarship. We draw on a multimodal ethnographic study with 36 young people aged 11–14 carried out over the course of one year at four UK-based informal STEM learning settings. Data collection included a series of interviews, observations and youth-created portfolios focused on STEM experiences. Illustrative case studies of two young men who took part in a community-based digital arts centre are discussed in detail through the theoretical lenses of Judith Butler’s *identity performativity* and Karen Barad’s *intra-action*.

**Results:**

We argue that physical and digital materiality mattered for the performances of ‘tech identity’ in that (i) the focus on the material changed our understanding of tech identity performances; (ii) digital spaces supported identity performances alongside, with and beyond physical bodies, and drew attention to new forms of identity recognition; (iii) identity performances across spaces were unpredictable and contained by the limits of material possibilities; and (iv) particular identity performances associated with technology were aligned with dominant enactments of masculinity and might thus be less accessible to some young people.

**Conclusion:**

We conclude the paper by suggesting that accounting for materiality in STEM identity research not only guides researchers in going beyond what participants say and are observed doing (and thus engendering richer insights), but also offers more equitable ways of enacting research. Further, we argue that more needs to be done to support the translation of identity resources across spaces, such as between experiences within informal and online spaces, on the one hand, and formal education, on the other.

## Introduction

Camera, phone, school, avatar, animation, animation speed, self-portrait, pride, frustration, pleasure, friendship, humour, traffic, weight, bulk, silence, talk, hesitations, absence, attendance, silver casing, black box, 500 Gigs SSD, terabytes, YouTube and selfies … perhaps all this stuff does not belong in the data, but I think we are missing something if it is left out. This is real and the stuff of young people’s lived experience of technology and STEM futures. (Data memo notes)

Identity has been of growing interest in STEM education research (Simpson & Bouhafa, 2020). Whether young people identify with science/STEM (e.g. see science as being ‘for me’, see themselves and are seen by others as a ‘science person’) has been found to relate to their aspirations, engagement and participation in science (e.g. Archer et al., 2013). Understanding how young people navigate their identity work is particularly important for addressing the persistent inequalities in science/STEM participation, which are shaped by gender, social class, ethnicity, dis/ability and other social axes. There is an abundance of evidence showing that it is easier for some young people to see themselves in science/STEM than it is for others, which has implications for their educational and professional trajectories, as well as for engaging with the subject(s) in everyday life (Carlone & Johnson, 2007; Godec, 2018; Gonsalves, 2014; Gonsalves, Silfver, Danielsson, & Berge, 2019; Kim, Sinatra, & Seyranian, 2018; Mendick, 2005; Rainey, Dancy, Mickelson, Stearns, & Moller, 2018; Vincent-Ruz & Schunn, 2018; Wong, 2016).

There has been an emphasis in STEM identity scholarship on discourse and, specifically, language. While there is a growing number of STEM identity studies (either relating to a specific subject like physics or considering the broader ‘STEM identity’), research findings have to date largely been based on the participants’ narrated accounts. Verbal and written articulations of identity have become a methodological norm, as evident from the dominance of studies utilising interviews, discussion groups and surveys (Varelas, 2012). Such privileging of language has been critiqued in the wider literature for possibly silencing the complexity of lived experiences, for it ‘fail[s] to fully account for the complex materiality of life’ (de Freitas & Curinga, 2015, p. 249). Barad (2007), whose work has guided the recent ‘new materialist’ movement in social research and some of whose concepts we utilise in this paper, has advocated for close reading of wide-ranging data, including the entanglement of language and other ‘things’. With seemingly polemical remarks like ‘[l]anguage has been granted too much power’ in social research (Barad, 2007, p. 132), Barad is problematizing the state of affairs where every ‘thing’, *even* materiality, is tied to language representations.

The focus on language arguably normalises and valorises a particular (classed, gendered and racialized) ability to ‘tell the self’ (Skeggs, 2004), while also providing limited opportunities for some younger participants and non-native speakers to express themselves. Research participants who might not possess specific resources of privilege to perform identity verbally might therefore risk being invisible, which raises concerns about the equity of research endeavours. To this point, we would add that not only does language have a somewhat privileged place in social research, but also *some* language/s and language forms tend to be more powerful and more privileged than others (e.g. Foucault, 1988; Hall & Gay, 1996).

We acknowledge that STEM identity research has not exclusively relied on language and recognise the important work that has employed broader methodological approaches. A number of researchers have, for example, focused on observations of embodied performances of identity (e.g. Archer et al., 2016; Danielsson, 2011; Dawson et al., 2020; Mendick, 2005). These studies have looked at the ways that participants perform their identities through physical appearance, behaviours and interactions. Attending to embodied enactments of identity has contributed rich insights about how some ways of being and doing were congruent with STEM, while others posed challenges for identity negotiations. The studies referred to here have provided valuable insights about how, for instance, performances of femininity (e.g. physical appearance and ‘typical’ feminine behaviours) tend to sit uneasily with the dominantly valued performances of STEM, with technology and engineering being particularly problematic (Danielsson, 2011).

While a growing body of STEM identity scholarship has considered embodied performances (i.e. focusing on the materiality of human bodies), we suggest that there is further opportunity to draw on new materialist insights and extend the focus beyond the body. In their edited book *Material Practice and Materiality: Too Long Ignored in Science Education*, Milne and Scantlebury (2019) have pointed out that the lack of attention to materiality is prevalent across science education research; the gap is thus not specific to science/STEM identity scholarship. While studies attending to non-human materiality have been scarce, we found a number of examples that showcase the potential of this direction (e.g. Calabrese Barton et al., 2013; Gonsalves & Danielsson, 2017; Talafian, Moy, Woodard, & Foster, 2019). Calabrese Barton et al. (2013), for instance, have utilised the concept of ‘identity artifacts’ (drawing on the work of Leander, 2002) to demonstrate how a material focus can enrich our understanding of science identity work. Calabrese Barton and her colleagues examined how young people’s various physical artefacts, such as an award-winning rocket or a poem, mediated their identity work in sometimes productive and other times constraining ways as the young people moved between different educational settings.

## Theoretical framework

In thinking about the role of materiality in young people’s identity performances, we draw on the conceptual work of Judith Butler (1990, 1993) and Karen Barad (2007). Despite some tension between the two scholars (see below), we suggest that bringing the two theoretical frameworks together is productive for including materiality. It is relevant to mention here that we did not start this piece of research by prior, foundational commitment to this specific conceptual orientation. Instead, we turned to Butler’s work on identity performance and Barad’s new materialism as a way to help us explain the repeated presence (and seeming importance) of materiality within young people’s STEM identifications that we observed in our data, as exemplified by the data memo notes that opened this paper. In this sense, we selected our theoretical framework in response to what we were noticing in the data. Yet, this does not mean that the process was solely data-driven and unidirectional. Rather, our process was guided by assemblages of ‘things’, as the research team mobilised theoretical resources, ethnographic accounts, case studies, photographs and field notes to develop articulations of the relations between human, material and conceptual phenomena. Next, we introduce our theoretical resources in more depth, highlighting in particular how these have dealt with materiality.

### Butler’s identity performativity

Butler (1990, 1993) has argued that identity is ‘performative’, a ‘doing’ rather than a ‘being’. She has pointed out that the performative ‘doing’ of identity depends on the particular discourses that are prevalent within a given space and the physical (material) bodies that enact these performances. For example, performative acts and their recognition are likely to differ in the context of mature women and that of queer boys. In her work, Butler (1993) has explicitly acknowledged the importance of materiality, writing, for example, that ‘[i]t must be possible to concede and affirm an array of “materialities” that pertain to the body’ (p. 66).

Despite considering materiality in her work, Butler has predominantly focused on embodiment (i.e. the materiality of human bodies) and paid less attention to the role of other, ‘non-human’ objects. This limitation of Butler’s performativity work has been noted by Barad:

Judith Butler’s performative account of mattering thinks the matter of materiality and signification together in their indissolubility; however, Butler’s concern is limited to the production of human bodies. (Barad, 2007, p. 145)

While some critique from new materialism scholars has perhaps been unjustified, such as that discursive work is essentially a-material (Barad, 2007), we agree that non-human materiality could be more extensively considered in discursive research (and in the specific area of science/STEM identity research, as we argued above).

### Barad’s concept of *intra-action*

While we regard Butler’s identity performativity lens compelling to think with, we agree that her work alone might ‘fail to provide an adequate account of the relationship between discursive practices and material phenomena’ (Barad, 2007, p. 146), particularly for thinking *beyond* embodied practices. Thus, we found Barad’s work on new materialism helpful for extending our thinking about the role of the matter in STEM identity work. We consider Barad’s concept of *intra-action* and her thinking about agency to provide useful insights in our theoretical framework. Barad has introduced the term *intra-action* as an alternative to ‘interaction’, which she has regarded as relating to pre-established bodies and ‘things’ that are in action with each other. She has used *intra-action* to instead emphasise that meaning and matter are essentially entangled.

For Barad, all phenomena are co-constructed enactments between human actors and non-human things (see also Latour, 2005; Law & Mol, 2002). In relation to this, Barad (2003) has advocated that agency is not an individual property, but rather ‘agency is a matter of intra-acting; it is an enactment, not something that someone or something has’ (Barad, 2007, p. 235). In other words, she has proposed that agency is located in the actions between material ‘things’, calling for particular attention to be paid to the said *intra-actions* between human and non-human entities. From this perspective, Barad has critiqued poststructuralist perspective on agency, arguing, for instance, that ‘for both Butler and Foucault, agency belongs only to the human domain’ (2007, p. 145). Barad’s notion of ‘distributed agency’ has been productively taken up in different ways in existing scholarship across various research domains, such as to explore performative pedagogies in getting university students to narrate the distributed agency of buildings (Mcphie, 2018) and to examine the power dynamics of the classroom (Murris, 2016).

Coming from a sociological perspective and taking a strong equitable stance in our own research work, we see Barad’s *intra-actions* as encompassing multiple entanglements between actors, objects and spaces, which are intertwined with power relations and related to the issues around im/possibilities. We argue that Barad’s work productively complements a Butlerian lens because, as Palmer (2011, p. 5) has noted in her work on mathematical subjectivities, Barad ‘sharpens the theoretical tool of [Butler’s] performativity’ through taking an interest in the *intra-activity* of matter in this process (see also Ringrose & Rawlings, 2015).

## Focus of this study

In the new materialism literature, the emphasis has largely been on the philosophical ‘mattering of matter’ and on theorising the relations between things human and non-human (Barad, 2007). We advocate that in a more practical sense, improving STEM participation and engagement, and indeed what counts as STEM, is tied to the progress in understanding the complexities of how particular STEM identities become and endure, or not. Considering the current science/STEM identity scholarship, we suggest that more attention might be usefully given to non-human materiality. With this paper, we thus seek to extend the current research by focusing on physical and digital materiality and its role in young people’s STEM identity negotiations. Specifically, we address the following research questions:

1. How do young people engage with physical and digital (non-human) materiality in ways that support, or not, their identity performances in relation to technology?
2. How does paying attention to materiality enrich our understanding of STEM identity work?

While within the wider project, we focus on STEM and locate our work within the wider science and STEM education research, the two case studies we discuss in depth in this paper predominantly relate to technology.

## Methods

### Data collection: multimodal portfolios and ethnographic field notes

In this paper, we draw on multimodal ethnographic data: field notes from ‘portfolio sessions’ (see below), multimodal portfolios, semi-structured and unstructured interviews, discussion groups and photographs of artefacts and spaces. Data were collected as part of Youth Equity + STEM (YESTEM) project that, broadly, aims to understand young people’s engagement with informal STEM learning and explore equitable practices within this space. We recruited 36 young people who voluntarily participated in programmes across four informal STEM learning settings; we worked with between six and 13 young people per setting. The selection criteria for recruitment into the study included age, gender, ethnicity, socio-economic background and the length of the young people’s involvement with the particular setting. Where possible, we were interested in recruiting young people with more substantive/longer-term involvement. Young people in this study were aged between 11 and 14, 22 were girls and 14 were boys, 16 self-identified as White British, seven as Black, two as Asian and the remaining 11 as mixed or other ethnicities.

The participants were initially approached to take part in the research by the educators working within the four organisations, and later met with a member of the research team who explained the nature of participation and ensured that the ethical guidelines were being met. The research project was presented to the young people as an opportunity to share views about their STEM engagement in and out of school, with the aim to help make STEM a more welcoming place for more young people like them. In addition, the research team emphasised to the young people that they were experts in their own lives and that understanding their experiences and perspectives would be highly valued and useful for informing future provision of STEM education. All the young people whom we invited to the study agreed to take part.

Young people were involved in the study as co-researchers, meaning that they participated in collecting and co-producing data (see examples of some artefacts they produced in the “Results and discussion” section). We organised between six and ten small-group sessions at each setting, where young people worked on constructing portfolios of their STEM-related experiences and discussed various aspects of their STEM engagement. These sessions were co-led by educators and researchers. The portfolio sessions involved, for instance, creating a self-portrait and documenting everyday STEM engagement (e.g. young people were asked to research and document their STEM experiences in their home, school and other settings).

During the portfolio sessions, we kept detailed field notes, took photographs and recorded conversations. We paid particular attention to what the young people were doing and how, when and where different materials were being mobilised in communicating with us and others. We also attended to how different physical and digital spaces supported or hindered STEM engagement and identity work, through investigating the social context and the specific material aspects, such as the availability of technological resources and access to additional communication channels (e.g. the online forums and social media). As researchers, we were entangled with the research process; we were not simply uninvolved observers, but took part in running the portfolio sessions where we interacted with the young people, such as through encouraging them and complimenting them on their artefacts.

During the portfolio sessions, we regularly engaged with young people in unstructured conversations and, after the sessions, conducted individual interviews following a semi-structured interview schedule. Questions most relevant to this paper included, for example: Would you say you are a science/tech person? Why, why not? What makes someone a science/tech person? Can you think of anyone who sees you as a science/tech person? Finally, we met young people for a follow-up discussion six months after the end of portfolio sessions. All interviews and discussion groups were audio recorded and transcribed. We combined individual and group data collection sessions in order to facilitate both peer discussions and provide space for young people to converse with us directly. The latter enabled some quieter and less confident participants to share their experiences in a different setting. The data that we ultimately co-constructed with participants varied across the cohort; in some cases, data for an individual participant consisted mostly of audio transcripts, with a few drawings or photographs to accompany them. In other cases, verbal and written accounts were minimal and the data were primarily in the form of digital and physical artefacts.

Our data collection approach was purposefully open and flexible, which was motivated by a growing body of literature advocating for the need to enable multiple modes of communication, particularly when working with younger participants and/or people who might struggle to articulate and present their thinking through language (Thomson, 2008). All young people we worked with in this study received a tablet or an action camera, which some used to take photos and videos to include in their portfolios (e.g. photos of science and technology in their lives). In some cases, additional technology and skills support was available to facilitate other techniques through which young people could perform themselves, such as digital drawing tools and stop motion animation.

The creation and interaction of various modes of communication were often synchronous. This meant that, for example, a young person might have been creating a product, such as an animation, while talking to us about a different aspect of their STEM involvement. Although material artefacts like drawings, photographs and other visual forms served as valuable prompts for conversations, we also thought of these as giving insight into richer, different meanings in and of themselves—and were especially valuable for young people who were less keen or comfortable expressing themselves through language. During the portfolio sessions, we observed the connectivity of various modes of communication and the ways young people interacted with the material/medium. The ‘mode’ of communication relates to the senses, so can be visual, linguistic, aural, spatial, gestural and so forth. At the same time, the term mode also has a lineage in computer design where the same actions produce different effects in different modes. The term ‘medium’ is linked to media (e.g. print, animation) and has a lineage in media studies heralded by McLuhan and Fiore’s (1967) famous book *The Medium is the Massage*. In our data, dis-aggregating mode and medium distracts from the focus on what is being mobilised and to what effect. To avoid linguistic gymnastics, we found ourselves conflating the terms mode and medium and in doing so, have come to recognise this as part of the process of analysis and writing *intra-action*.

### Data analysis

We started data analysis by reading the data within the research team. We looked at how young people ‘narrated the self’ (verbally performed their identity), i.e. how they talked about their identity in relation to STEM. This generated accounts of how identity was discursively performed through language (Butler, 1990, 1993) and involved how young people spoke about science/tech/STEM as being for them or not, the recognition they received from others, and what it meant to be a science/tech/STEM person. Our analytical process was iterative; the *assemblage* (Barad, 2007) included data, theory and concepts that we found to be useful for shedding the light on the role of materiality in STEM identity research.

Next, we moved to mapping out how the material conditions shaped identity work by looking at the ‘material moments’ (Taylor, 2013), i.e. moments that we interpreted as signifying *intra-action* between our participants and the digital and physical materiality. Following Barad, we engaged in close reading of relational effects, such as how things were connected and emerged as important. This meant paying attention to *intra-action* dynamics of where, when, who and what that was inclusive of language, academic text and the researcher, while being mindful of the power and privilege of particular types of data. The analysis involved a close reading of the rich ethnographic accounts generated by the researchers and data in photographs and portfolios from the young people. We attended to what digital and physical matter the young people shared with us and/or mobilised during the sessions, and how these examples were then actively referenced or used.

Inspired by other researchers, we also explored the affective relations within the data and selected particular segments that ‘glowed’ at us to examine further (MacLure, 2013); this process informed our case study selection. For the segments that ‘glowed’ (i.e. appeared particularly interesting or significant for answering our research questions), we mapped out the collection of things human and non-human that were making specific enactments possible. This inferencing went beyond the dialogic analysis; it was integrated into the process as a series of accounts that were in effect descriptions in the spirit of ‘look what is going on here’.

**Fig. 1:**
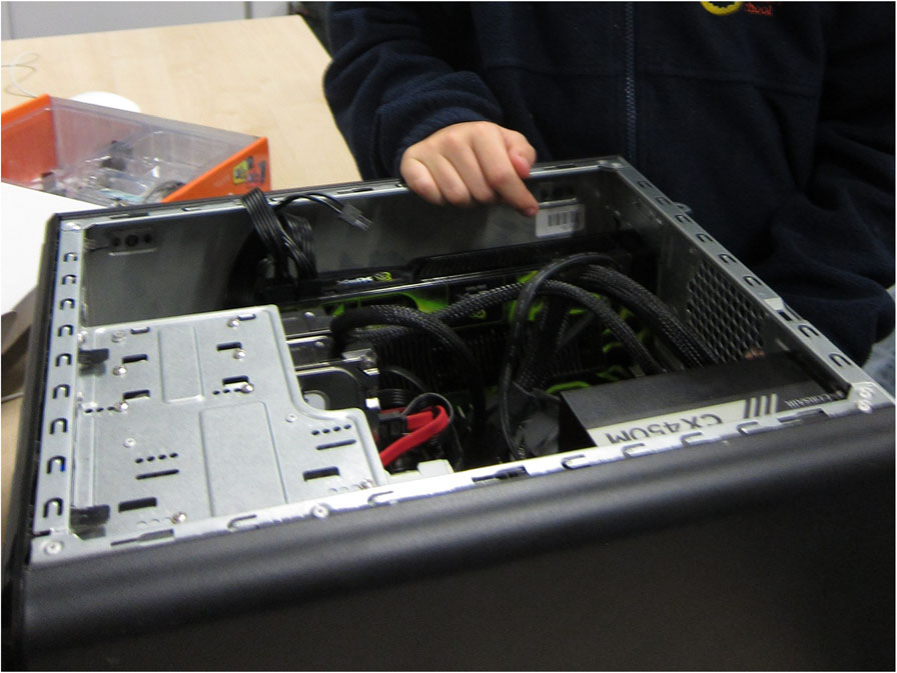
Black-and-White’s home-built computer

The analysis involved a critical reading of what role different physical, digital and social spaces had in supporting or hindering young people’s identity performances. Particular attention was paid to the material-discursive *intra-actions* where the material came into play in relation to recognition, competence and self-identification. This involved, for instance, how the young people used material objects to perform their identity in relation to technology. We would suggest that the physical presence of material objects, for example, a home-built computer (see Fig. 1), was vital for making the specific *intra-action* possible. It was the physical presence of this object that facilitated Black-and-White’s prolonged explanation of the computer parts and capabilities, which we interpreted as enactments of tech identity performance. Such rich enactment, we suggest, would be impossible without the specific material object. Thus, in our analysis, we paid close attention to what was being gathered at any specific moment in time, e.g. the researcher, the educator, the portfolio and the technology-rich educational space.

Some of the questions that guided our analytic process included the following: What materials seemed to be essential to a particular STEM identity performance? What made specific enactments possible or impossible? Where and how were specific identity performances viable? Where did they appear to stumble, falter or be dismissed? What were the relational shifts over time and space? Following the analysis, we reflected our interpretation back to the young people and invited comment and critique. This was done through creating a printed booklet for each young person that included our analysis about their STEM experiences, identity and aspirations, along with the various data we collected (e.g. photos from the portfolio sessions, quotes from the interviews). As part of the interviews, young people were encouraged to offer their own interpretations and challenge us on our ongoing analysis presented in the booklet.

### Selecting the case studies

Attending to materiality seemed to be particularly valuable in the case of young people who felt less comfortable or less keen on expressing themselves verbally or in writing. The accounts of these young people may have been missed had we taken a more traditional qualitative research approach that relied on language as the primary mode of expression. For instance, when we asked the young people about their engagement and identification with science and technology (e.g. would you say you were a science/tech person? Why/not?), some found their answers difficult to articulate (see Ginger’s example below).

For this paper, we selected two young men (Ginger and Black-and-White, both 11 years old; pseudonyms were chosen by the participants) from our larger study to present in greater depth. Having reviewed all the cases, these two appeared to be the clearest and strongest examples of how non-human materiality mattered for their tech identity performances, as well as being particularly interesting in terms of illustrating what might have been invisible or anomalous if we focused in our research only on language. Both young men found expressing themselves verbally and/or in writing challenging in different ways (e.g. Black-and-White asked us to avoid writing and Ginger’s responses to our questions were mostly monosyllabic, as he admitted that ‘it’s quite hard for me to answer these questions’; he preferred to write ‘when I type it I’m just so much better at it’). At the same time, these two young men produced rich and meaningful data through other forms of self-expression during portfolio sessions. A further reason for selecting these two participants was that the setting within which this study was located (a community-based digital arts centre) appeared to have played a particularly important role in their identity negotiations. We also found different and complex manifestations of the two young men’s identity work in other spaces, such as at home and at school, which made them particularly interesting to focus on.

## Results and discussion

### Reading material-discursive entanglements as identity performances

We start by presenting two vignettes of ‘material moments’ (Taylor, 2013) that stood out to us as particularly interesting in terms of materiality being intimately related with the young men’s identity performances and recognition. During one of the portfolio sessions, Black-and-White brought along a home-built computer (see Fig. 1), telling us that the computer showed ‘the tech I do in my life’ and explained with technical terminology what the significant features were. The field notes below summarise the occasion.

We arrive at the centre early and wait in the lobby for the participants to arrive. The first to come are brothers Spuggs and Black-and-White. Black-and-White is carrying a large black box, which I notice is computer hardware. As we inquire what this is (and why he brought this with him – it looks heavy!), he responds that it is to show us what he built at home. During the portfolio session, Black-and-White is not keen to get involved with making an animation, which is an optional activity today. Instead, he chats to us about the computer. He insists that we make sure to record all specifications correctly. He dictates us to write: ‘there is a lot of memory in there, two terabytes, 500 Gig SSD, that’s a lot of memory’, pointing to the various components that are the memory as he talks. He visibly labours across the room with the computer that he’s built, refuses help, and puts it under the camera (which others are using to make animation) so he can take a picture of it for the project portfolio. (Constructed from field notes, November 2017)

We suggest that Black-and-White’s display of his home-built computer can be interpreted as a performance of his skills and knowledge related to technology. He had briefly spoken to us previously about his involvement with technology, such as that he was the fastest in the weekly sessions to assemble robots. This point that was also recognised by others within the programme at the time, e.g. loud comments made by the educator during the weekly sessions ‘Black-and-White in the corner is our tiddlybot expert’ (‘tiddlybot’ is a Raspberry Pi robot kit). Notably, it was evident that the materiality of the computer further supported and was a key part of Black-and-White’s performance of tech identity. The *intra-action* between the non-human (home-built computer) and human (Black-and-White) was enacted with pride, respect and recognition—we speculate that in the absence of the computer, the enactment may have taken a different form, perhaps one that offered a more limited insight to Black-and-White’s performance of tech identity.

We suggest that the hand on the computer case in Fig. 1 might give some indication of the physical effort involved in Black-and-White’s ‘owning’ the casement of the computer and the delicate attention to detail within the limited space of the casing. He engaged with a female researcher in a masculine demonstration of power and pride (akin to the performance of ‘muscular intellect’, involving a confident display of expertise, see Archer et al., 2017; Mac An Ghaill, 1994). Black-and-White’s expertise appeared to be vital in how he negotiated competence and identity work; ‘I know I’m good at building things because I am. I build PCs’. His involvement with technology also featured in his future aspirations; he hoped to ‘build PCs’ when he got older, intimating the importance of physical materiality for this continuous, long-term engagement with technology.

**Fig. 2:**
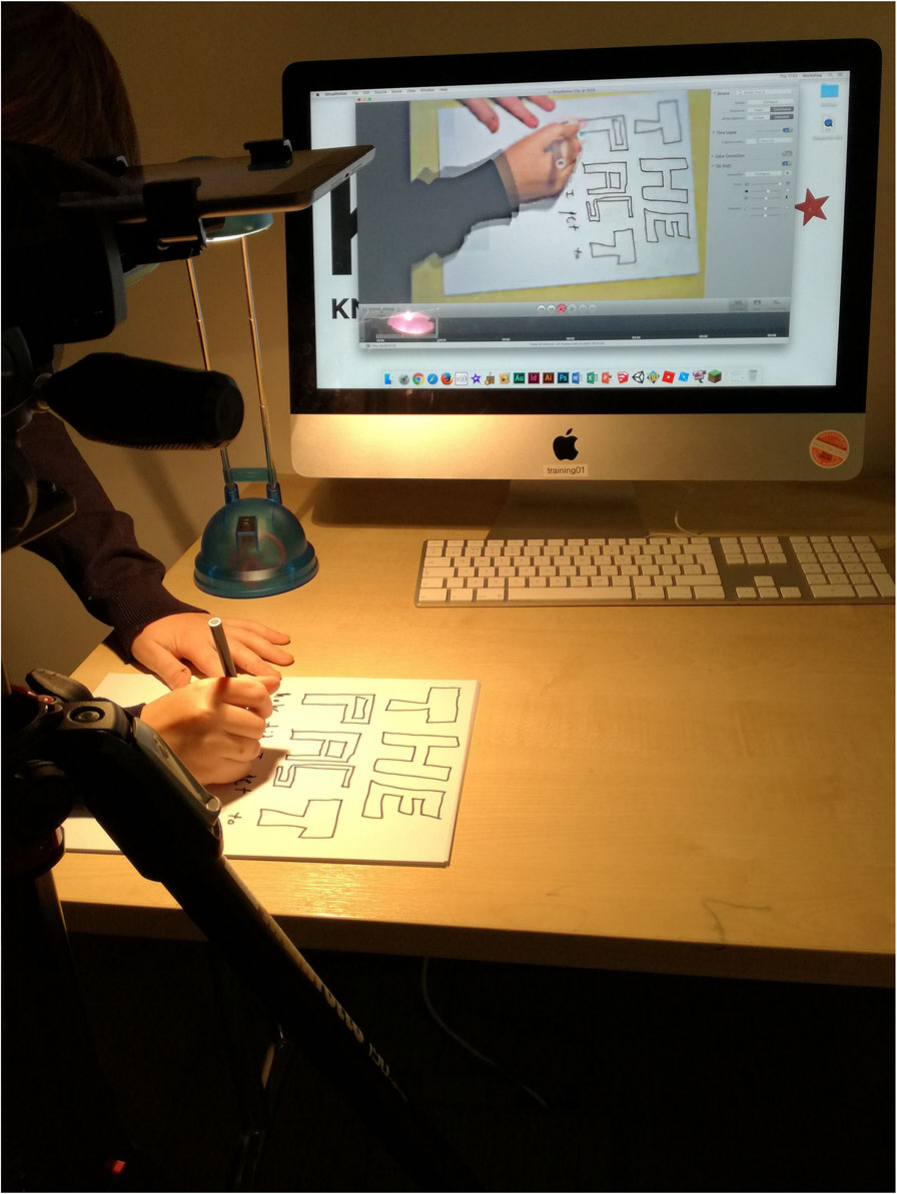
Ginger’s stop motion animation

Ginger’s modality of choice, on the other hand, was digital. During one of the portfolio sessions, he created an elaborate stop motion animation (see Fig. 2), assembling his drawings and writings together in a short animation about ‘my life in STEM’.

In the computer room, four ‘animation stations’ are set up – one for each participant, should the boys choose to create an animation as a way of communicating their STEM engagement (not everyone decides to). The practitioner gives a brief introduction, i.e. that they could focus on their journey through STEM, but encourages creativity. Ginger sets up his station (an iPad facing down, recording the images) and starts drawing and writing. He does not require any assistance. He seems to be in the flow and we don’t interrupt. This is his first time using this specific technology setup and software, and we comment a couple of times how quickly he is picking up the skills. Ginger makes an impressive 30-page animation, drawing and writing non-stop for 90 minutes. The animation shows a story of how he first found out about the community centre, got into coding, what he has been doing since, and how these activities related to his future aspirations and life outside. (Constructed from field notes, November 2017)

Figure 2 illustrates the complexity of the *intra-action*: the elements of light, camera focused on a drawing pad, action on the surface that is being digitised on a screen. Later, these images and words were manipulated into a timeline to communicate Ginger’s STEM journey. At the time the image was captured, Ginger was not particularly interested in talking to the researcher but nor was he indifferent to showing us what he knew, thought about and was able to do, using his preferred mode of communication. The functionality of the software and input devices appeared to be as important to Ginger’s identity performance as the content; virtual space was brought into physical space as a way of communicating. The animation provided a medium through which Ginger was comfortable expressing his thoughts and experiences. We suggest that there were additional possibilities that became evident through the conceptual lens of *intra-action*, which enabled a display of skills through the creation of a digital artefact. The meaningful modality was a digitised product, which supported identity performance beyond words and beyond the traditional form of a pen-and-paper.

For both Ginger and Black-and-White, materiality mattered in a sense of being intimately entangled in the production of their tech identity; we argue that it was not separate and not just a vehicle. Digital and physical materials were ‘vital players’ (Bennett, 2010) in how the two young men produced their identities. Both were making choices in terms of how to communicate. These choices included not just content but also the medium, i.e. multimodal options as they choose between available material and linguistic resources to construct and animate (in some cases, literally) their tech identity. As Miller (2010, p. 60) has argued, ‘objects make us, as part of the very same process by which we make them’. The mode of presentation in the cases was deeply entangled with technology. Through creating and interacting with the technology, Ginger and Black-and-White were constructing and performing their STEM identity in ways that language alone may not have supported. Their agency was emergent from *intra-action* with matter, which was central to being recognised for their expertise and contributing to their identity performance (Carlone & Johnson, 2007).

### Beyond physical bodies and spaces—digital spaces and identity recognition

Spaces mattered for negotiation of tech identity performances. For Ginger, the *intra-actions* with the digital, online space seemed to play a particularly important role in his identity work (see Leonardi (2010) for a similar discussion). Ginger would often share with us the digital products he created outside the project sessions (e.g. online games, which led to chats with other gamers). He also had several online channels where he would post his work, which he spoke about with pride (‘I just share it with the whole entire world’; ‘I get people from far away following me!’).

Ginger’s tech involvement appeared to be closely connected to his future aspirations; he told us that he wanted to be a games designer and added ‘I already am, really’, highlighting his experience with online games design. We argue that Ginger’s identity work appeared to be performed largely through, and in turn enhanced by, the digital medium where he regularly gained recognition for his work (‘people think because of how advanced my games are that I’m much older’), indicating the importance of the particular space in shaping identity performance. In this way, we suggest that his identity performance occurred in relation with the online community. As others have pointed out, online involvement includes performative acts in and of themselves, providing powerful tools and a space to perform and develop tech identity (Cover, 2012).

Ginger’s enactment of identity in digital spaces can be seen as going beyond the confines of both his physical body and his physical location. The expanded *intra-actions* of Ginger’s identity performance can also be interpreted as enabling translocational and transformational identity work (Anthias, 2002, 2008). Anthias (2008) has, for instance, proposed that different spaces (and times) matter for people’s identity work, as people are inevitably positioned differently. Ginger’s engagement with the digital world could, therefore, be interpreted as a form of such translocation (i.e. being positioned differently in different spaces), enabling him to perform expertise and be recognised in ways that supported his present and future identity work. In this way, the material aspect of space (in this instance, digital) played a crucial role in Ginger’s performance and recognition of tech identity.

### Limited transferability of tech identity performances across spaces

It is well established that identity performances shift and change across space and time and depend on which enactments are ‘intelligible’ (Butler, 1993), supported and recognised within particular contexts (for some examples in science education, see Archer et al., 2017; Carlone, Scott, & Lowder, 2014). In this study, materiality played a role in supporting, and sometimes hindering, tech identity work as the two young men moved across different physical and digital spaces.

Ginger and Black-and-White both appeared to successfully perform tech identity in some spaces (namely, the informal STEM learning setting, to some extent at home and in Ginger’s case, additionally, in the online space). The physical environment of the community-based digital arts centre, the technology available there and the support and recognition the young men received from their peers and educators played an important role in their tech identity performances. However, these successful performances appeared to be limited to a specific time and space or what could be interpreted as an example of Barad’s (2007) ‘timespacemattering’. For Ginger, digital performances (e.g. his coding and games design, with associated recognition) seemed to only partly translate into the spaces of home and school. For instance, he would regularly speak about not being able to engage with technology at school (‘they don’t really care about it at our school’), displaying a strong critique of the absence of opportunities:

They want to encourage people to do tech and be a tech person but then in school, it’s not an option to do ICT [Information and Communication Technology] until you’re year 8, literally halfway through year 8! School is definitely not ready for the internet age! (Ginger, interview)

Ginger also admitted that he rarely talked about technology at home, commenting that his family found his involvement with technology ‘a bit odd’ (or what might in Butler’s terms be labelled ‘unintelligible’) and his parents did not understand much of what he is doing; ‘sometimes my dad helps me come up with ideas for the games, but that’s pretty much it, really’. Black-and-White, similarly, commented that he got little recognition for his technical skills at school, where access to technology was limited (‘laptops at school don’t even work’; ‘computers are rubbish’). The availability of technology was crucial for both Ginger’s and Black-and-White’s engagement and performances of expertise and identity. Its absence, likewise, hindered opportunities for tech identity performativity and recognition.

The young men were able to perform tech identity within the informal STEM learning setting, but less so at school. The disjuncture between the identity enactments that were possible or impossible in particular spaces appeared to have implications for the young men’s tech/STEM engagement and raised a concern about their future trajectories. Ginger, for instance, spoke about starting to doubt his abilities for the future: ‘I’ll do whatever the school thinks I’m capable of doing’; ‘I might be a coder, I don’t really know, depends on what people say I could do’.

The inconsistencies of opportunities for identity enactment, we suggest, were experienced by the young people as troubling. We interpreted Ginger’s statements as possibly complicating his tech identity—Ginger struggled to see his future self in technology. Similarly, we suggest that Black-and-White’s difficulties at school and the lack of opportunities to engage with technology there made it challenging for him to perform tech identity in ways he did within the informal STEM learning setting. The sense of powerlessness, we suggest, raises concerns about the resilience and longevity of tech identity performances when these are weakly supported within the mainstream educational spaces. The findings indicate that physical and digital spaces, time and matter, might enable the longer-term potential for these performances to be sustained (and possibly consolidated into STEM trajectories). The young men’s performances were ‘read’ differently in different spaces, such as by their parents and their teachers, who the young men intimated did not extensively recognise and support their involvements. In the same way as being positively recognised across multiple spaces might amplify tech identity work, dissonance between spaces is likely to cancel out the positive effects from one space as a person moves to another.

### Were technology-supported tech identity performances open to everyone?

The young men’s performances of technical knowledge and mastery/expertise, such as demonstrated by Black-and-White’s technical explanations and *intra-actions* with the computer he brought in to show us, and by Ginger’s frequent references to specificities of his coding work, could be interpreted as exemplifying dominant alignments with masculinity. Similar dominant associations of computing, coding and tech with masculinity have been widely noted in the literature (Francis, 2000) and have been regarded as reproducing exclusivity and stereotypical associations of technology and tech identities (Charles & Thébaud, 2018). We suggest that these ways of performing tech identity might risk reinforcing popular geeky, a-social notions of who participates in technology and who performs tech identities, which in turn restricts the extent to which tech identity might be open to other youth (Varma, 2007, 2010).

Equity issues around who might be able to perform technology-supported tech identity performances were raised across our wider study cohort. For instance, we offered all young people in our study (*n* = 36) the use of multimodal, technology-enhanced tools to author their identities (e.g. providing tablets and action cameras), but comparatively few did so. This was especially telling for the group of young women who took part in an all-women STEM club that included coding events, given the programme’s explicit focus on technology. These young women appeared to prefer verbal, pen-and-paper and photographic modes to construct their portfolios. They tended to use tablets mostly for selfies, performing identity through social media and digital spaces, which we suggest tended to be social rather than tech identity performances (Dawson et al., 2020). While we do not seek to draw broader conclusions from the small numbers of young people we worked with, we suggest that the young people’s tendency to perform identity through different modalities can be interpreted as both reflecting and (re)producing dominant gender relations. That is, it was the masculine enactments that seemed to most successfully mobilise the space, time and material resources to perform recognisable tech identities. In turn, such performances were largely absent within data on young women in our study.

## Conclusions

In this paper, we reported on a study of young people’s tech identity performances, drawing on conceptual tools of *identity performativity* (Butler, 1990, 1993) and *intra-action* (Barad, 2007). In our data collection, we encouraged multiple forms of expression, which was especially valuable for young people who found it challenging to engage in verbal performances of identity through interviews and written forms. Including the material in the research process supported performances of young people’s interest and expertise, which we argue was critical for supporting identity negotiations for a more diverse range of young people. Had we only used more traditional language-based and observational research methods without specifically enabling and including the material entities, some of the young people’s identity performances may have been less visible, or misread as ‘thinner’ or invisible.

This paper seeks to make original empirical and methodological contributions to STEM education research. Empirically, the paper contributes to new understandings of identity performativity through exploring the role of materiality in the production of tech identity performances. We interpreted the material-discursive entanglements as performances of tech identity that can be read and interpreted similarly to verbal or written forms and in ways that expanded our understanding. Matter not only served as a mode of expression, but was also intimately connected to how young men like Ginger and Black-and-White performed identity. Moreover, the case studies demonstrated that both digital and physical materiality were integral to how the two young men produced their identities, highlighting the role of both tangible and intangible non-human matter. While STEM education literature has previously, at least to an extent, considered the role played by physical objects in young people’s identity work (e.g. Calabrese Barton et al., 2013), our findings suggest that future research would usefully benefit from extending the focus also to non-tangible, digital materiality, especially given the role that technology and the internet play in young people’s lives. We found that Barad’s *intra-action* shifted and disrupted the direction of analysis by enriching what was possible to articulate and claim.

The research suggested that identity enactments did not always travel easily across spaces. Previous research has similarly found that identity work is often context-specific; identity performances enacted in one space do not necessarily extend to another. Several scholars have, for instance, previously pointed out the disparities of opportunities for science/STEM identity performance between informal learning spaces and the school science classroom (Calabrese Barton et al., 2013; Thompson, 2014), or even between two different science classrooms (Carlone et al., 2014). We found that STEM identity performances across different (physical and digital) spaces were also influenced by the material opportunities available to young people, which we suggest adds a valuable, novel perspective to existing scholarship. The weakened opportunities for tech identity performances at school, such as a lack of equipment and adequate support, posed challenges to the sustainability of identity work. Experiences in informal spaces and online worlds that enabled and supported Ginger’s identity work—and we would say, were crucial for his engagement with technology—were at risk of being ‘cancelled out’ at school, threatening his longer-term identity work and trajectory.

Methodologically, accounting for non-human materiality offers a way to study tech identity performances that goes beyond the focus on what youth say and what researchers observe. We argue that this approach offers more scope to those who might find verbal articulation difficult. As others have proposed, the reliance on language-based articulations tends to privilege particular people/modalities/data (Milne & Scantlebury, 2019; Skeggs, 2004). As Mazzei and McCoy (2010, p. 515) remind us, to move the research endeavours forward, we need to open up what ‘counts’ as data in order to give voice to diverse participants and diverse forms of expressions. Moreover, this approach led us to consider agency within the specific enactments, not solely as a property of an individual, thereby emphasising the importance of looking at phenomena through human/non-human *intra-actions* and paying sufficient attention to materiality.

Based on our findings, we would call for more focus on multimodality in STEM identity research. We argue that accounting for various forms of materiality might offer a less deficit, more equitable way to study a wider range of tech identity performances, enabling enactments that are owned and celebrated by young people who might otherwise risk being missed or side-lined. Broadening the approach to studying STEM identity performances can help highlight equity and inclusion challenges facing young people in maintaining, protecting and developing STEM identities while moving between digital and physical spaces of home, school, informal educational spaces and the online. Importantly, our approach enabled us to gain insights that may have been masked otherwise.

We anticipate that increased attention to materiality and multimodality could also be beneficial to informal STEM learning practice, such as for moving endeavours towards greater openness, sensitivity and responsiveness to what is going on with young people. This focus might entail supporting diverse ways of communicating, engaging with STEM and performing identity, along with recognising how the presence and absence of particular ‘matter’ might advance or curtail engagement opportunities. Further, supporting multimodality might facilitate more equitable practice where young people (and particularly those that may have been marginalised within dominant educational spaces) might be able to leverage ways of performing STEM identity—not only through verbal and written performances, but also through their engagement with physical and digital materiality.

Finally, we would suggest that more needs to be done to support the translation of identity resources across spaces, such as through closer collaboration between science education providers and greater recognition of the varied forms of STEM engagement. The lack of scope for Ginger and Black-and-White to perform valued tech identities across different spaces points to disjunctures between identity performances that demonstrate experiences, skills and knowledge, and limited recognition for such performances in the school context. The findings of this study highlight the importance of alternative provision for engaging with STEM. While it might be difficult to effect change within families and the support available to young people within their home environment, the findings of this study suggest that connectivity between formal and informal educational spheres could be improved to better support young people. Existing literature offers insights into identity development in relation to technology, including within digital spaces (Bennett, Maton, & Kervin, 2008; Goode, 2010); it would be valuable to consider how such endeavours might best be supported in other educational spaces. To conclude, the study presented in this paper provides valuable evidence that more needs to be done to recognise and value diverse young people’s skills, experiences and modes of identity work within dominant spaces, such as within the formal education system, in order to support them in the ongoing development of their identity work in relation to STEM.

## Data Availability

For ethical and data protection reasons, we are unable to share any data from this project.

## Abbreviations

STEM: Science, technology, engineering and mathematics
